# Neurodevelopmental regression, severe generalized dystonia, and metabolic acidosis caused by *POLR3A* mutations

**DOI:** 10.1212/NXG.0000000000000521

**Published:** 2020-10-07

**Authors:** Vanessa Zanette, Aurelio Reyes, Mark Johnson, Daniel do Valle, Alan J. Robinson, Vaneisse Monteiro, Bruno Augusto Telles, Ricardo L.R. Souza, Mara L S.F. Santos, Cristiane Benincá, Massimo Zeviani

**Affiliations:** From the Medical Research Council—Mitochondrial Biology Unit (A.R., M.J., A.J.R., C.B., M.Z.), University of Cambridge, United Kingdom; Department of Genetics (V.Z., R.L.R.S., C.B.), Federal University of Paraná—UFPR; and Neuropediatric Division (V.M., M.L.S.F.S., D.V., B.A.T.), Hospital Pequeno Príncipe, Curitiba, Brazil.

## Abstract

**Objective:**

To expand the clinical phenotype of *POLR3A* mutations by assessing the functional consequences of a missense and a splicing acceptor mutation.

**Methods:**

We performed whole-exome sequencing for identification of likely pathogenic mutations in a 9-year-old female patient with severe generalized dystonia, metabolic acidosis, leukocytosis, hypotonia, and dysphagia. Brain MRI showed basal ganglia atrophy and presence of lactate and lipid peaks by [^1^H]-magnetic resonance spectroscopy. Expression levels of Pol III target genes were measured by quantitative real-time (qRT)-PCR to study the pathogenicity of the biallelic mutations in patient fibroblasts.

**Results:**

The patient is a compound heterozygous for a novel missense c.3721G>A (p.Val1241Met) and the splicing region c.1771-6C>G mutation in *POLR3A*, the gene coding for the catalytic subunit of RNA polymerase III (Pol III). Aberrant splicing was observed for the c.1771-6C>G mutation. Decreased RNA expression levels of Pol III targets (HNRNPH2, ubiquitin B, lactotransferrin, and HSP90AA1) were observed in patient fibroblasts with rescue to normal levels by overexpression of the wild-type protein but not by the p.Val1241Met variant.

**Conclusions:**

Mutations in the *POLR3A* gene cause *POLR3A*-related hypomyelinating leukodystrophy with or without oligodontia or hypogonadotropic hypogonadism (HLD7, OMIM: 607694) and neonatal progeroid syndrome (OMIM: 264090), both with high phenotypic variability. We demonstrated the pathogenicity of c.1771-6C>G and c.3721G>A mutations causing an early-onset disorder. The phenotype of our patient expands the clinical presentation of *POLR3A*-related mutations and suggests a new classification that we propose designating as Neurodevelopmental Disorder with Regression, Abnormal Movements, and Increased Lactate.

*POLR3A* encodes the catalytic subunit A of Pol III, which is responsible for the constitutive transcription of transfer ribonucleic acids, mitochondrial RNA–processing RNA, 5S, H1, and noncoding RNAs (ncRNAs), and involved in translation of several mRNA.^1,2^ Because of its involvement in the transcription of so many different RNAs, it is not surprising that mutations in *POLR3A* can lead to a variety of phenotypes.

Two different conditions are associated with *POLR3A* mutations: HLD7 (OMIM: 607694) and neonatal progeroid syndrome (NPS, OMIM: 264090). HLD7 is an autosomal recessive early-onset leukodystrophy^3^ presented by hypomyelination, hypogonadotropic hypogonadism, hypodontia, spasticity, dystonia, and neurodevelopmental regression.^1,4^

Like HLD7, NPS is also an autosomal recessive early-onset disorder presenting a wide range of phenotypes including macrocephaly, progeroid appearance, triangular face, micrognathia, nystagmus, hypodontia, muscle atrophy, hypertonia, and agenesis of the corpus callosum.^5^

Here, we present the c.3721G>A and c.1771-6C>G mutations in *POLR3A*, with features not described in HLD7 nor NPS. We propose that these mutations extend the phenotype of POLR3A deficiency and create a new classification of patients with features similar to those presented here.

## Methods

### Standard protocol approvals, registrations, and patient consents

Informed consent was obtained according to the Ethical Standards Committee of UFPR (CAAE: 84773818.2.0000.0102).

### Case report

The proband is a 9-year-old girl with a healthy mother who had no other pregnancies and a father diagnosed with depression. Gastroesophageal reflux and ineffective breast sucking were observed just after birth. When aged 5 months, she was apathetic and presented generalized dystonia. Four months later, she refused food with no swallowing disorder. Protein malnutrition was observed, and oral administration of hypercaloric diet was started with ensuing of metabolic acidosis (lactate 4.88, reference: 0.63–2.44 mmol/L, normal pH and decreased HCO_3_ 21.4 reference: 22–29 mEq/L). She could sit but neither crawl nor walk.

Brain MRI, muscle biopsy, and karyotype were normal at age 1 year. Improvement of weight gain and hydration was achieved by nasogastric tube feeding. Gastrostomy was performed but showed dumping, requiring thickening of diet.

She presented with recurrent lung infections, milestone regression, and was unable to talk at 2 years. Worsening of dystonia and hypodontia was observed, with the absence of 2 baby teeth, but permanent dentition developed.

**Figure 1:**
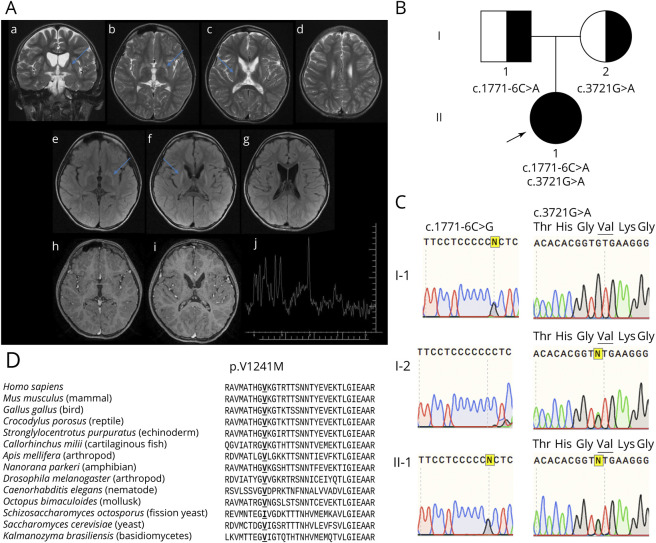
Brain MRI and chromatograms of DNA sequence and alignment of POLR3A in several species (A) Brain MRI of the proband showing hyperintense signal in T2 (blue arrows), volumetric reduction of the putamen and caudate nucleus (a and f). Spectroscopy (j) shows peak of lactate and lipids. (B) Segregation of *POLR3A* mutations c.1771-6C>A from the father and c.3721G>A from the mother to the proband (indicated by an arrow). (C) Chromatograms showing mutations c.1771-6C>A in heterozygosity in the father (I-1) and c.3721G>A (p.V1241M) in heterozygosity in the mother (I-2) and the presence of both in the proband (II-1). (D) Alignment of 1,241 residue of the POLR3A, species were selected from sequences obtained from a 4-iteration PSI-BlastP with default parameter research.

Another MRI was performed at age 4 years (data not shown) with total brain volume reduction and alterations in striatal bodies. Four years later, new MRI showed bilateral hyperintensity in T2 (figure 1A, a–d) and FLAIR (figure 1A, e–g), atrophy of the caudate nucleus and putamen with compensatory enlargement of the lateral ventricles. Absent enhancement on postcontrast images (figure 1A, h–i) and increase of lipids/lactate on [^1^H]-magnetic resonance spectroscopy were observed, but the noisy background could contribute to possible artifactual peaks (figure 1A, j).

EEG showed slight diffuse disorganization in background activity, reflecting a diffuse cerebral dysfunction, with no association with specific pathologic features. No epileptiform activity was observed.

Amino acid analysis in blood showed increased levels of threonine, glycine, alanine, alpha-aminobutyric acid, valine, cystine, isoleucine, leucine, tyrosine, phenylalanine, and ornithine. Urine amino acid analysis showed increased levels of 3-hydroxybutyric and reduced levels of methylsuccinic, 3-methylglutaconic, and 3-hydroxy-3-methylglutaric. In addition, she presents leukocytosis and persistent metabolic acidosis with increased lactate (8.5, reference: 0.6–2.2 mmol/L), decreased pH (7.12, reference: 7.32 to 7.43), and increased ammonia levels (47, reference: 11–32 µmol/L). Currently, she presents hypotonia, dysphagia, low weight, diffuse muscular hypotrophy, and severe generalized dystonia with worsening of the condition at night, but no triggers identified. Anticholinergic, benzodiazepine, antidopaminergic, gabaergic, and anticonvulsant medications were used to decrease dystonia, unsuccessfully. Physical neurologic examination shows orofacial dyskinesias, normal cranial circumference, and symmetry with neither abnormalities of cranial nerves nor facial dysmorphism. The patient is fully wheelchair dependent for ambulation.

### Analysis of WES data

DNA was extracted from blood (DNeasy Kit; Qiagen). Whole-exome sequencing (WES) was prepared (Illumina® Nextera) and run (HiSeq 2000). Human GRCh37 reference genome and a GATK-based pipeline^6^ were used. Sanger sequencing was performed by Source BioScience, United Kingdom.

### Fibroblasts

Fibroblasts were derived from the patient's skin biopsy and grown (high-glucose Dulbecco's Modified Eagle Medium Glutamax, sodium pyruvate, 10% fetal bovine serum, and penicillin/streptomycin) at 37°C (5% CO_2_). Cell immortalization and rescue experiments were performed by lentiviral transduction (pLOX-Ttag-iresTK, Addgene #12246, pWPXLd-POLR3A-HA “WT or V1241M”and “empty vector”).

For Western blot, cells were lysed (Tris-HCl 20 mM, NaCl 150 mM, EDTA 1 mM, Triton-X-100 1%, glycerol 10%, and MgCl_2_ 1.5 mM plus protease inhibitors), centrifuged, and supernatants mixed with loading buffer for sodium dodecyl sulfate-polyacrylamide gel electrophoresis (SDS-PAGE) and immunoblotting. Protein concentration was measured (DC protein assay; Bio-Rad).

### qRT-PCR

RNA was extracted using the Invitrogen TRIzol kit. cDNA was prepared with reverse transcriptase (Invitrogen). For POLR3A mRNA processing, cDNA was amplified by PCR, separated by agarose gel electrophoresis, gel extracted, and Sanger sequenced. qRT-PCR of target genes was performed in duplicate with Life Technologies TaqMan Assays (Applied Biosystems): ncRNA (HNRNPH2, Hs01395062_m1) and mRNAs: ubiquitin B (UBB), lactotransferrin (LTF), and HSP90AA1.

### Statistical analysis

The differences among groups were calculated with 95% confidence intervals (*p* < 0.05) using the one-way analysis of variance and Tukey multiple comparison test. All data are presented as mean ± SD of the mean (SD) of 3 biological independent experiments in duplicates.

### Data availability

Data will be available upon request.

## Results

WES detected 2 mutations in *POLR3A*, a missense c.3721G>A (p.Val1241Met–rs886141646), inherited from the mother, and a splicing region c.1771-6C>G (rs115020338), from the father (figure 1B–C). Sequencing of mtDNA (data not shown) revealed the presence of MT-CYB (c.14831G>A); no other rare variant of uncertain significance (VUS) was found.

The c.3721G>A mutation has a frequency of <0.0001 (TOPMed), and MutationTaster2 classifies it as possibly pathogenic (score 0.99). The valine residue in the 1,241 position is highly conserved in metazoans with bilateral symmetry, except in some species, which present isoleucine instead (figure 1D).

**Figure 2:**
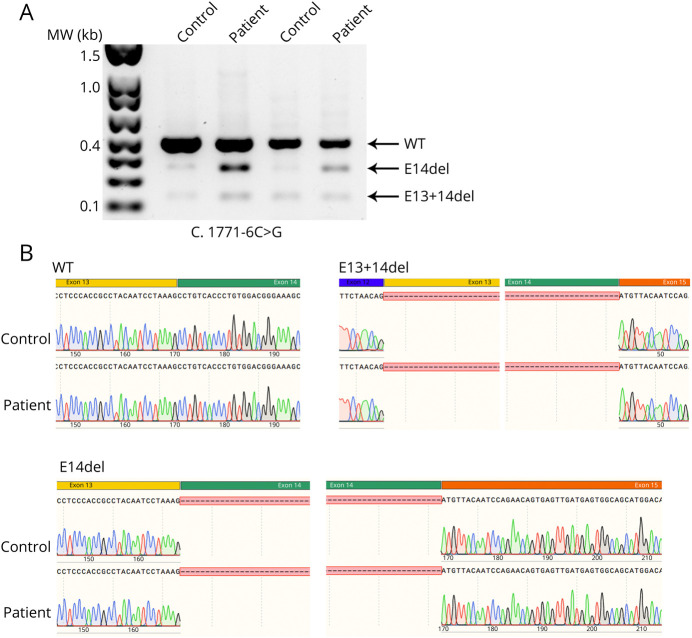
POLR3A transcript visualization and sequencing from patient and control fibroblasts (A) Agarose gel electrophoresis performed from cDNA extracted and amplified by PCR from control and proband (II-1) fibroblasts. (B) Sequencing of the *POLR3A* gene from wild-type (WT) and proband fibroblast demonstrating the deletion of exon 14 (e14del) and exons 13 + 14 (e13 + 14del).

Electrophoresis from control and proband fibroblasts detected 2 transcripts of shorter length along with the canonical full-length mRNA (figure 2A). The longest product corresponded to the full-length mRNA, the one right below it showed deletion of exon 14, whereas the smallest one displayed the combined deletion of both exons 13 + 14 (figure 2B). An accumulation of shorter compared with full-length products was more evident in the patient cells.

**Figure 3:**
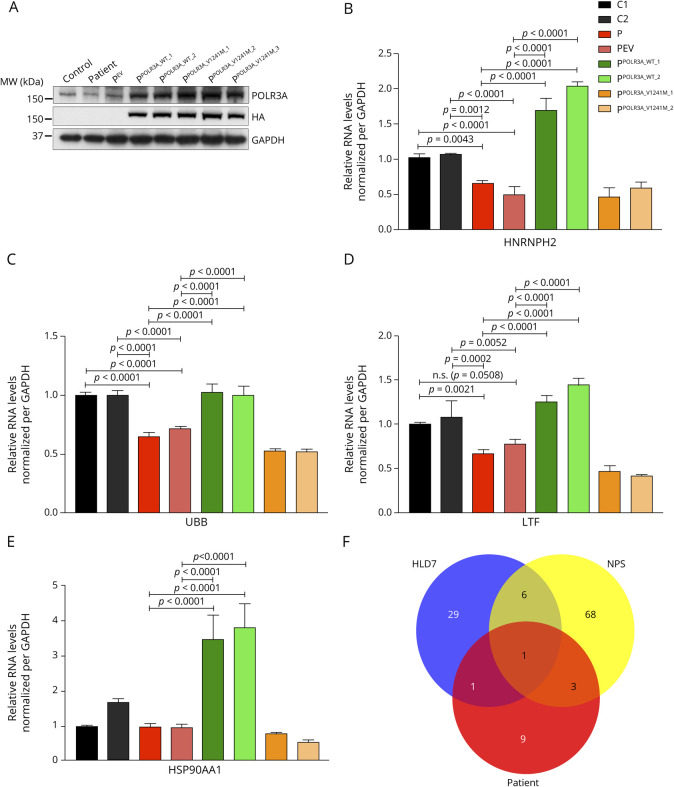
Western blot analysis of POLR3A and RNA expression levels of POL III targets in patient fibroblasts (A) SDS-PAGE for POLR3A, HA, and GAPDH in control (Ctrl) and proband (P) fibroblasts expressing empty vector (P^EV^), POLR3A-HA wild type (P^POLR3A_WT_1^ and P^POLR3A_WT_2^), and POLR3A-HA mutant (P^POLR3A_V1241M_1^, P^POLR3A_V1241M_2^, and P^POLR3A_V1241M_3^); qRT-PCR measurement in fibroblasts isolated RNA showing levels of (B) HNRNPH2, (C) UBB, (D) LTF, and (E) HSP90AA1 normalized per GAPDH. (F) Intersection among the patient features (caudate nucleus atrophy, dysphagia, dystonia, finger clubbing, hypodontia, hypotonia, lactate peak, leukocytosis, lipid peak, low weight, metabolic acidosis, milestone regression, putamen atrophy, and recurrent respiratory infection) and symptoms described for NPS (aged face, agenesis of the corpus callosum, apparent macrocephaly, ataxia, beak-shaped nose, blue sclerae, broad eyebrows, Chiari malformation, cryptorchidism, Dandy-Walker malformation, decreased subcutaneous fat, deep-set eyes, dental delayed eruption, developmental delay, downslanting palpebral fissures, downturned corners of mouth, ectropion, endocrine abnormalities, entropion, failure to thrive, fat accumulation around the buttocks, dysphagia, frontal bossing, generalized lipoatrophy, gynecomastia, hydrocephalus, hypertelorism, hypertonia, hypodontia, hypoplasia of the facial bones, hypoplastic ilia, hypotonia, hypotrichosis, increased triglycerides, intention tremor, intrauterine growth retardation, irregular metaphyseal endplates, joint contractures, lagophthalmos, large feet, large hands, long fingers, long thin bones with enlarged metaphyseal endplates, long toes, lower eyelid covering part of the cornea, malar hypoplasia, mandibular hypoplasia, mental retardation, micrognathia, muscle atrophy, nasal high-pitched voice, natal teeth, nystagmus, parietal bossing, partly unossified atlas at birth, persistent fontanelles, pinched nose, pointed chin, poor postnatal growth, progeroid appearance, prominent abdomen, prominent forehead, prominent scalp veins, pseudohydrocephalus, recurrent respiratory infections, scoliosis, short stature, small mouth, sparse eyebrows, sparse eyelashes, thin diaphyses, thin ribs, thin translucent skin, thin upper vermilion, triangular face, trident configuration of acetabula, upslanting palpebral fissures, and widely open sutures); and HLD7 (abnormal smooth pursuit, ataxia, bladder dysfunction, cerebellar atrophy, cerebellar signs, cognitive regression, cortical atrophy, decreased vibratory and positional sense, delayed dentition, dental delayed eruption, delayed puberty, developmental delay, drooling, dysarthria, dysmetria, dysphagia, dystonia, extensor plantar responses, hyperreflexia, hypodontia, hypogonadotropic, hypogonadism hypomyelination, leukodystrophy, motor regression, myopia, nystagmus, oligodontia, optic atrophy, peripheral neuropathy, postural tremor, seizures, short stature, spasticity, thinning of the corpus callosum, upper motor neuron signs, vertical gaze limitation, and white matter rarefaction).

We tested the dependency of the patient's fibroblast phenotype on defective POLR3A by expressing HA-tagged POLR3A, either wild type (P^POLR3A_WT_1^ and P^POLR3A_WT_2^) or mutant (P^POLR3A_V1241M_1^, P^POLR3A_V1241M_2^, and P^POLR3A_V1241M_3^), using the empty vector (P^EV^) as a negative control. Compared with the control (Ctrl), patient-derived cell lines (P and P^EV^) displayed low levels of POLR3A, whereas overexpressing cell lines presented higher levels of the protein, as also demonstrated by anti-HA antibody immunovisualization (figure 3A).

A significant decrease in the levels of the HNRNPH2, UBB, LTF, and HSP90AA1 was observed in the patient's fibroblasts compared with controls in all cases except for HSP90AA1 compared with one of the controls, C1 (figure 3B–E). As expected, no significant difference was detected between P cells and P^EV^. Furthermore, patient cells overexpressing the wild-type protein recovered basal or higher levels of Pol III target genes. This was not the case for patient cells overexpressing the p.V1241M variant, as their levels were similar to or even lower than both patient cell lines, P and P^EV^.

## Discussion

Here, we present a case report of a compound heterozygous for c.1771-6C>G and c.3721G>A mutations in *POLR3A.* The patient presented clinical features associated with HLD7 and NPS, but also metabolic acidosis, leukocytosis, lipid and lactate peak.

Leukodystrophy, characterized by hypomyelination or demyelination,^7^ hypogonadotropic hypogonadism, and nystagmus were absent, suggesting that the diagnosis of HLD7 is not appropriate. Features associated with NPS, but not with HLD7, such as feeding difficulties, recurrent respiratory infections, and hypotonia, were identified in our patient. Nevertheless, classical features of NPS such as progeroid appearance and malar hypoplasia were not present, and diagnosis of NPS may also be unsuitable. Hypodontia is the only symptom shared between our patient and both described syndromes, whereas dystonia is presented only in HLD7 (figure 3F).

The c.1771-6C>G mutation is located 6 nucleotides upstream exon 14 of *POLR3A,* resulting in the loss of this exon. It is classified as a VUS (ClinVar), and it has been reported in a family with HLD7, causing basal ganglia atrophy with no hypomyelination.^3^

Loss of exon 14 has been linked to reduced expression of the splicing factor HNRNPH2, responsible for the regulation of pre-mRNA splicing.^3^ Expression levels of HNRNPH2 were reduced in patient's fibroblasts, and we showed that overexpression of WT POLR3A, but not of the mutated p.Val1241Met variant, rescued the expression of HNRNPH2. Decreased expression of UBB, LTF, and HSP90AA1 has been previously found in other patients carrying the c.1771-6C>G mutation in *POLR3A*.^3^ All the mRNAs expression levels were decreased in the patient cells, with rescue of their expression levels when WT but not p.Val1241Met POLR3A was overexpressed, demonstrating the pathogenicity of the c.3721G>A mutation.

No other VUS with correlation between the patient's phenotype and previously associated disease genes was identified in the nuclear genome. In mtDNA, the mutation m.14831G>A was found in a patient with Leber hereditary optic neuropathy, but no functional studies were performed.^9^ This variant was recently classified as benign in ClinVar following the American College of Medical Genetics guidelines^10^ (evidence codes: BS1, BS2, and BP4).

The clinical phenotype of our patient resembles a combination of symptoms of 2 conditions associated with *POLR3A* mutations and even mutations in genes coding for POL III targets,^8^ highlighting the difficulty of a clinical diagnosis and reinforcing the importance of a detailed clinical report combined with molecular studies.

In conclusion, we suggest a new classification to patients carrying mutations in *POLR3A,* with neither leukodystrophy nor NPS features, which we propose designating as Neurodevelopmental Disorder with Regression, Abnormal Movements, and Increased Lactate.

## Glossary

ncRNA: noncoding RNA
NPS: neonatal progeroid syndrome
qRT: quantitative real-time
SDS-PAGE: sodium dodecyl sulfate-polyacrylamide gel electrophoresis
VUS: variant of uncertain significance
WES: whole-exome sequencing
